# Empirical substitution models of protein evolution: database, relationships, and modeling considerations

**DOI:** 10.1093/database/baaf052

**Published:** 2025-09-24

**Authors:** Paula Iglesias-Rivas, Roberto Del Amparo, Javier A Cabaleiro, Miguel Arenas

**Affiliations:** CINBIO, Universidade de Vigo, 36310 Vigo, Spain; Department of Biochemistry, Genetics and Immunology, Universidade de Vigo, 36310 Vigo, Spain; CINBIO, Universidade de Vigo, 36310 Vigo, Spain; Department of Biochemistry, Genetics and Immunology, Universidade de Vigo, 36310 Vigo, Spain; CINBIO, Universidade de Vigo, 36310 Vigo, Spain; CINBIO, Universidade de Vigo, 36310 Vigo, Spain; Department of Biochemistry, Genetics and Immunology, Universidade de Vigo, 36310 Vigo, Spain

## Abstract

Substitution models of protein evolution describe the patterns of amino acid substitutions over evolutionary time and are fundamental for probabilistic methods of phylogenetic inference. At the protein level, a variety of substitution models are available, but only empirical substitution models are well established in phylogenetics due to their mathematical simplicity. Despite their importance, a database compiling the large number of currently available empirical substitution models of protein evolution is lacking, although such a resource could facilitate access, assessment, and subsequent implementation of these models into phylogenetic frameworks. Besides, little is known about formal comparisons between the current set of empirical substitution models. We present *EModelDB*, a database of empirical substitution models of protein evolution required for probabilistic protein phylogenetics that includes the corresponding exchangeability matrices, model classification, and model-specific biological information. The database is integrated into a graphical user interface, written in Python and SQL, that facilitates its usability. We also compared common empirical substitution models in terms of the distance between their relative rates of amino acid substitution and amino frequencies at equilibrium. We found that substitution models derived from proteins related in nature tend to cluster together, reflecting similar evolutionary patterns. Indeed, we evaluated the empirical substitution models in terms of the folding stability of the derived modeled proteins and found that they generally produce less stable proteins compared to real proteins, suggesting that substitution models with additional evolutionary constraints can be preferred for studying protein evolution accounting for folding stability.

**Database URL**: https://github.com/Paula-Iglesias-Rivas/EModelDB

## Introduction

Substitution models of evolution are required for diverse probabilistic evolutionary analyses of molecular data such as phylogenetic tree and ancestral sequence reconstructions [1–6], estimation of evolutionary parameters [7, 8], detection of molecular adaptation [9, 10], and selection among evolutionary scenarios [11], among others. A variety of substitution models are available at the nucleotide, codon, and amino acid levels [12].

It is known that different substitution models can yield varying phylogenetic likelihoods when applied to a particular dataset [13, 14]. However, there is a debate about the influence of substitution model selection on the accuracy of phylogenetic inferences. Some studies found that applying the best-fitting substitution model based on likelihood does not affect the accuracy of phylogenetic tree reconstruction [15, 16] or ancestral sequence reconstruction [17]. However, other studies found that applying the best-fitting substitution model does have some effects, albeit small, on phylogenetic tree reconstruction and ancestral sequence reconstruction [4, 5, 7, 18, 19]. Our previous studies indicated that the effect of the selected substitution model on the accuracy of phylogenetic inference is influenced by the diversity of the dataset under study [4, 5]. If the dataset has low genetic diversity, model selection becomes less important because the evolutionary trajectories are clearly defined, and the model does not have to make crucial decisions about possible past evolutionary states. By contrast, if the dataset has high genetic diversity, model selection plays a more important role in determining past evolutionary states, especially among states with similar probabilities under neutral evolution. Altogether, selecting and applying an appropriate substitution model by default for analyzing a particular dataset is recommended [20–22].

At the nucleotide level, the traditional substitution models comprise a few parameters (i.e. transition/transversion ratio and nucleotide frequencies) that are usually estimated from the study data [20, 23]. However, at the protein level, the traditional substitution models are empirical because they include a large number of parameters that must be estimated from protein databases [12, 24]. Additional substitution models of protein evolution were developed (i.e. structurally constrained substitution models [12]) but, despite their high accuracy [1, 19, 25], they are not yet well established in phylogenetics due to their overall complexity in implementation and computational requirements. In contrast, empirical substitution models are well established in the field due to their mathematical simplicity, despite relying on unrealistic assumptions such as site-independent evolution and the use of the same evolutionary patterns across all protein sites.

Empirical substitution models of protein evolution generally include models based on substitution rate matrices and models based on score matrices. The models based on substitution rate matrices (*M*) describe the instantaneous relative rates of change between each pair of amino acids (i.e. from *i* to *j*) *M_ij_* and, in most cases, include amino acid frequencies at equilibrium (*f*) [12]. These matrices, together with amino acid frequencies at equilibrium when available, are traditionally used in phylogenetics to obtain exchangeability matrices (*Q_ij_ = M_ij_f_j_*) and subsequent probability matrices of substitution events between amino acid pairs over a certain evolutionary time period (*t*, i.e. a branch length in a phylogenetic tree) [$P( t ) = {{e}^{Qt}}$], which are essential for likelihood-based phylogenetic inferences [26]. On the other hand, the models based on score matrices provide observed substitution scores for amino acid changes and are traditionally used in sequence alignment algorithms [27, 28].

As previously indicated, the parameters of the empirical substitution models are usually estimated from large amounts of protein sequences to consider as much evolutionary information as possible and this estimation is implemented in some computer frameworks [10, 29]. More than 100 empirical substitution models of protein evolution (including models based on instantaneous rate and score matrices) are available to date, derived from different taxonomic groups and protein families, including nuclear proteins (e.g. [30, 31]), chloroplast proteins (e.g. [32]), mitochondrial proteins (e.g. [33, 34]), and viral proteins (e.g. [35–37]), among others. As expected, having a wide variety of empirical substitution models of protein evolution is beneficial because they provide different fits for specific real data (i.e. viral protein data usually fit better with empirical models based on viral proteins than with empirical models based on other proteins [35, 36]) and, therefore, phylogenetic frameworks should incorporate as many empirical substitution models as possible to allow users to apply an appropriate model for the study data. Considering the growing number of available empirical substitution models of protein evolution and their convenient implementation into phylogenetic frameworks, we noticed that a database compiling empirical substitution models of protein evolution could be useful to the field. Thus, we present a database of empirical substitution models of protein evolution that, for each model, includes the matrix of relative substitution rates or scores among amino acids, amino acid frequencies at equilibrium, classification into taxonomic or protein family groups, references, and other relevant information. The database is implemented into an intuitive graphical user interface (GUI) written in Python and SQL, can be easily updated to incorporate new models, and is freely available at https://github.com/Paula-Iglesias-Rivas/EModelDB. Next, we investigated the distances among the main empirical substitution models of protein evolution, in terms of relative rates of change among amino acids and amino acid frequencies at equilibrium, to evaluate whether closely related models are derived from similar proteins. We also evaluated the accuracy of the main empirical substitution models in modeling protein folding stability.

## Materials and methods

### Implementation of the database of empirical substitution models of protein evolution

The database includes all the currently available empirical substitution models of protein evolution, to our knowledge. Although it is primarily focused on substitution models based on symmetric matrices of relative substitution rates traditionally used in phylogenetics, it also includes substitution models based on score matrices, which, as indicated, are useful for building multiple sequence alignments. The database was implemented into the *EModelDB* framework, which is written in Python using the SQLite3 library (SQLite Consortium) to provide an optimized structure for efficient queries and data access. Indeed, it includes a user-friendly GUI, also written in Python using the Streamlit library and Cascading Style Sheets for styling. The GUI is directly connected to the SQLite database, thus facilitating fast and reliable data access.

**Figure 1.**
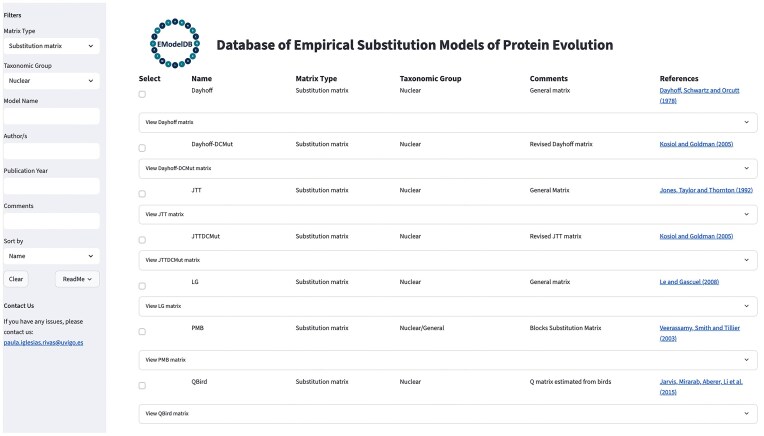
Illustrative caption of the GUI of the database of empirical substitution models of protein evolution. The figure shows the GUI with a filter applied to select empirical substitution models for nuclear proteins that can be used for probabilistic phylogenetic inference methods. On the left side, the GUI displays a list of available filters that can be applied, along with contact information for users to submit suggestions, new models, or provide feedback to the administrator. Users can select the desired substitution models and download the corresponding matrices of relative substitution rates among amino acids and the amino acid frequencies at equilibrium.

The framework enables the direct visualization of the substitution models and allows filtering by phylogenetic (instantaneous rate) or score matrices, taxonomic groups and protein families, publication year, authors, and other criteria (Fig. 1). Users can also sort the substitution models by the specified criteria using a dropdown menu. Additionally, the database includes informative comments and references for each substitution model. The selected substitution models can be downloaded individually or as a group. Next, the relative rates of change or scores among amino acids and amino acid frequencies at equilibrium of the substitution models can be downloaded as text files in the traditional *PAML* format [10, 38, 39], which is commonly used in phylogenetic frameworks. The framework includes contact information to support updates of the database, allowing for the incorporation of new substitution models and revisions to existing content. These proposals will be evaluated as part of a necessary procedure to ensure proper and accurate updates to the database. Additional information about the framework and its GUI is provided in Fig. 1. The framework is freely available at https://github.com/Paula-Iglesias-Rivas/EModelDB and is distributed with detailed documentation for installation and execution. In addition, the database can be used remotely, allowing users to explore the models online at https://emodeldb.streamlit.app/. We conducted rigorous query tests, selecting and downloading substitution models of specified taxonomic groups, to verify the integrity and functionality of the database.

### Clustering of empirical substitution models of protein evolution

A variety of empirical substitution models are available for phylogenetic inference, but it is unclear whether their similarity in terms of exchangeability matrix correlates with their similarity in terms of taxonomic groups (e.g. viral proteins, mitochondrial proteins, nuclear proteins, and general proteins, among others). We explored this aspect by calculating distances, based on relative substitution rates among amino acids, amino acid frequencies at equilibrium, and a combination of both, for all pairs of common empirical substitution models that can represent the taxonomic groups present in the database. To perform this comparison, we normalized all substitution rate matrices by dividing each rate of change by the highest rate within the respective matrix, as amino acid substitution rate scales can vary across models. The distances between each pair of substitution rate matrices and/or amino acid frequency vectors were calculated as the mean of the absolute differences between corresponding substitution rates or amino acid frequencies. Next, we identified the relationships among the empiricalsubstitution models of protein evolution using a bottom-up agglomerative clustering method (i.e. neighbor joining [40]) based on the specified distances among the studied empirical substitution models.

### Evaluation of folding stability of proteins modeled with empirical substitution models

As previously indicated, empirical substitution models of protein evolution are widely used in phylogenetics despite certain unrealistic assumptions. For example, these models have often been used to reconstruct ancestral protein sequences [41, 42]. However, little is known about the folding stability of proteins modeled with empirical substitution models compared to the folding stability of real proteins. We investigated this aspect by simulating protein evolution under different empirical substitution models, followed by the prediction and comparison of the folding stability of the simulated proteins with that of real proteins. We studied eight datasets, representative of different protein families, available from the PFAM database and following a previous study on protein evolution [43]. The datasets presented a different number of sequences, sequence length, sequence identity, and a representative protein structure of the corresponding protein family provided by PFAM (Table 1). For each dataset, we identified the best-fitting empirical substitution model of protein evolution, according to the Bayesian Information Criterion following a previous study [44], using the *ModelTest-NG* framework [23] (Table 1). Next, for each dataset, we inferred a maximum likelihood phylogenetic tree using the *RAxML-NG* framework [45], with outgroups indicated in a previous study [43], and under the selected substitution model. Given the phylogenetic tree, the best-fitting substitution model (Table 1), and the representative protein structure of the dataset, we performed a simulation of protein evolution from the root to the tip nodes of the phylogenetic tree under the corresponding best-fitting substitution model with the framework *ProteinEvolver* [46]. We also simulated data under the corresponding best-fitting substitution model but ignoring the heterogeneity of the substitution rate among sites, as well as under other closely related and distant empirical substitution models, to explore potential influences of different substitution models on the folding stability of the modeled proteins. We assumed the sequence of the representative protein structure as the sequence of the root node, considering it as representative of the study data. For each study dataset and substitution model, the simulation was performed 100 times to account for the stochasticity of the Markov substitution process. Finally, we predicted the folding stability of the real and simulated protein sequences, using the representative PDB structure as structural template and replacing indels to avoid biases in the structural prediction, with the framework *Prot_evol* [19, 43, 47, 48].

**Table 1.** Study data from different protein families

Protein family	PFAM code	Number of sequences	Sequence length	Sequence identity	Representative PDB code	Empirical substitution model
DNA ligase	PF13298	124	128	0.59	3P4H	WAG + I + G
Ferredoxin	PF05996	25	242	0.51	3NB8	LG + G
Heat shock protein	PF00012	32	600	0.50	2KHO	LG + G
Kinesin	PF00225	85	323	0.39	3KAR	LG + G
Oxysterol-binding protein	PF01237	26	436	0.52	1ZHT	LG + G
Retroviral aspartyl protease	PF00077	18	112	0.45	3OGQ	rtREV + G
Rubredoxin	PF00301	39	53	0.53	1R0F	WAG + I + G
Triosephosphate isomerase	PF00121	53	236	0.37	1TTI	LG + G

For each data, the table presents the PFAM code, the number of sequences, sequence length, sequence identity (mean of pairwise sequences), a representative protein structure (PDB code) associated to the corresponding protein family, and the identified best-fitting empirical substitution model of protein evolution according to the Bayesian Information Criterion. “+I” refers to the consideration of a proportion of invariable sites and “+G” refers to the consideration of variation of the substitution rate among sites according to a Gamma distribution.

## Results

### The implemented database of empirical substitution models of protein evolution

The database includes all currently available empirical substitution models of protein evolution, to our knowledge, comprising 61 empirical (rate) substitution models and 69 score models. It can be accessed through the GUI, which provides user-friendly exploration, filtering, and download of the data. In particular, the user can group and filter models according to diverse criteria, and download the score matrix or the matrix of relative substitution rates among amino acids and amino acid frequencies at equilibrium for the selected models. The models were carefully reviewed, and the database will be updated, after evaluation by the authors, with new substitution models or proposed modifications. The GUI is available on GitHub, along with detailed information for installation and execution, and we successfully tested it on various operating systems, including Linux, macOS, and Windows.

### The substitution patterns described by empirical substitution models of protein evolution are related to the taxonomic groups of the underlying proteins

**Figure 2.**
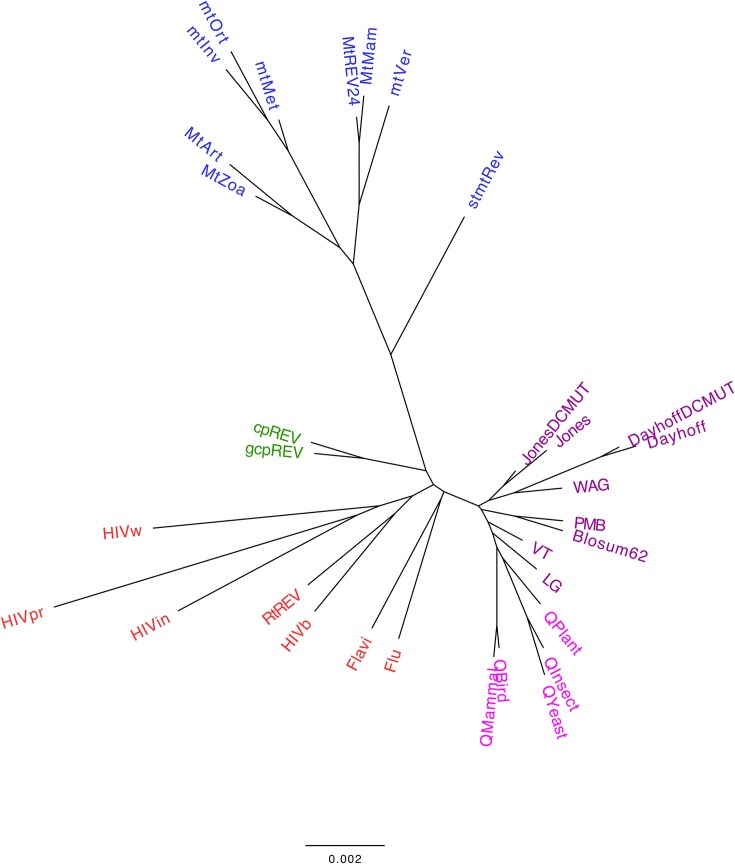
Agglomerative clustering of common empirical substitution models of protein evolution according to the matrix of relative substitution rates among amino acids and the amino acid frequencies at equilibrium. The normalized matrix of relative substitution rates among amino acids and the amino acid frequencies at equilibrium, with equal contribution, were applied to determine the distance between every pair of commonly used substitution models. A bottom-up agglomerative clustering method, neighbor joining, was then applied. Each model was color-coded based on the type of proteins used to construct the model: mitochondrial proteins (blue), chloroplast proteins (green), virus proteins (red), and nuclear proteins (general in purple and taxon-specific in pink). Clusters based on the matrix of relative substitution rates among amino acids or amino acid frequencies at equilibrium are presented in Supplementary Figure S1 and Supplementary Figure S2 (Supplementary Material), respectively.

Next, we explored whether phylogenetic empirical substitution models derived from closely related taxonomic groups also present similar distances in amino acid substitution rates, as reflected by the distances between their exchangeability matrices. The clustering analysis, based on the distance among the main empirical substitution models and considering the matrix of relative substitution rates among amino acids and amino acid frequencies at the equilibrium, showed a grouping of the models that generally aligns with the biological nature of the proteins used to construct the corresponding models (i.e. mitochondrial proteins, chloroplast proteins, virus proteins, and nuclear proteins; Fig. 2). This finding indicates that the substitution process varies among proteins from different taxonomic groups, which may evolve under specific selection pressures. It also supports previous studies that indicated the importance of selecting an appropriate substitution model for the evolutionary analyses of particular protein data [4, 5, 13, 49, 50]. When the matrix of relative substitution rates among amino acids and the amino acid frequencies at equilibrium are evaluated separately, the grouping is less clear (Supplementary Figure S1 and Supplementary Figure S2, Supplementary Material), as expected, due to the reduced evolutionary information being considered. This clustering provides a global overview of current empirical substitution models of protein evolution. Additionally, considering the currently available substitution models, we noticed that further substitution models of protein evolution are needed to model the evolution of other proteins (i.e. those from other viruses or distinct proteins or protein domains within the same virus [35, 51]).

### All the empirical substitution models produce unrealistically unstable proteins although the best-fitting empirical substitution model partially reduces this bias in some protein families

**Figure 3.**
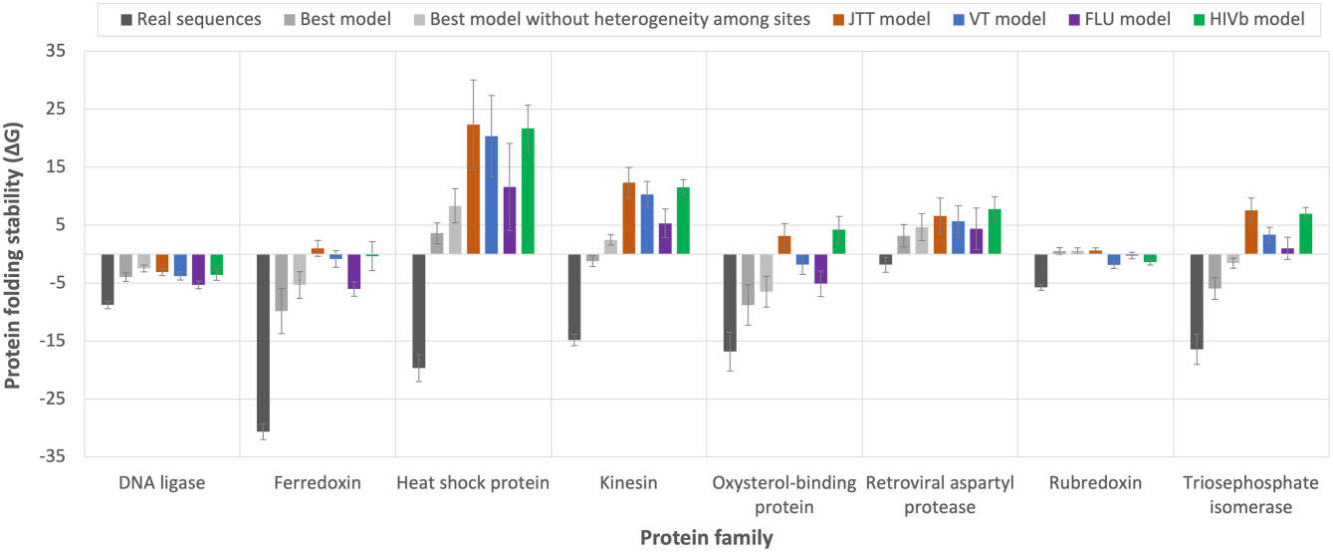
Protein folding stability predicted in real and simulated protein sequences from different protein families. For each protein family, the folding stability of the real protein sequences is shown as a reference (dark gray). The folding stability of protein sequences simulated under each empirical substitution model is shown in different colors. The error bars indicate the 95% confidence interval among sequences of the multiple sequence alignments. In general, real protein sequences exhibit higher stability compared to those simulated under any empirical substitution model. Interestingly, in general, the best-fitting empirical substitution model tends to produce the most realistic folding stabilities.

Empirical substitution models of protein evolution are traditionally used to reconstruct ancestral protein sequences [41, 42]. Since these models make several assumptions, such as maintaining the same exchangeability matrix among protein sites or ignoring direct evolutionary constraints from the protein structure, the stability of the derived modeled proteins could be affected. To assess this influence in different empirical substitution models, including the best-fitting substitution model obtained with traditional likelihood-based approaches, we conducted a simulation study. In particular, for different protein families, we considered a phylogenetic tree derived from real protein data of the studied family and a representative protein structure from that family to simulate protein evolution along the phylogeny under various empirical substitution models of protein evolution, including the best-fitting empirical substitution model selected using traditional phylogenetic likelihood. The results indicated that all the empirical substitution models produce protein sequences that are less stable than the real protein sequences (Fig. 3). Interestingly, for several protein families (i.e. Ferredoxin, Kinesin, and Triosephosphate isomerase), the best-fitting empirical substitution model partially reduced this bias, producing proteins that were slightly more stable than those obtained with other substitution models. Another interesting finding was that removing the variability of the substitution rate among protein sites, which is present in all the best-fitting empirical substitution models (Table 1), resulted in less stable proteins (Fig. 3). This suggests that accounting for this heterogeneity in the substitution rate among sites is important for obtaining proteins with more realistic stability.

## Discussion

Phylogenetic methods based on probabilistic approaches (i.e. maximum likelihood and Bayesian inference) require substitution models of molecular evolution to be incorporated into their likelihood functions [26]. At the protein level, empirical substitution models based on instantaneous rate matrices are well established in the field due to their mathematical simplicity (i.e. they often assume reversibility of the substitution process), which facilitates their straightforward incorporation into likelihood functions. Similarly, empirical substitution models based on score matrices are also well established in methods for multiple sequence alignment [27, 28]. Thus, a variety of empirical substitution models of protein evolution have been developed using large databases of proteins from certain taxonomic groups or protein families. These models are particularly valuable for the community when they are accessible and integrated into analytical frameworks. We identified a need for a compendium of all currently available empirical substitution models, as it could facilitate their finding and subsequent implementation into analytical frameworks, among other applications. Thus, we present a database of empirical substitution models of protein evolution that, to our knowledge, formally comprises all currently available empirical substitution models (including their matrices of relative substitution rates among amino acids, score matrices, and amino acid frequencies at equilibrium). The database can be explored through a user-friendly GUI, which provides a variety of information for each model and allows users to select the models according to various criteria. Additionally, it can be easily updated with new models or modifications upon request.

Next, we explored whether the similarity of the models in terms of the distance between their exchangeability matrices aligns with the similarity of the models based on the nature of their underlying proteins. We observed this relationship where, e.g., models based on viral proteins group together, as do models for mitochondrial and chloroplast proteins, among others, when all the evolutionary information provided by the models is considered. Naturally, with less information, this grouping is reduced. These findings suggest that proteins from different taxonomic groups evolved under different selection pressures, which has important practical implications for phylogenetic analyses. In particular, it highlights the importance of using at least a model derived from proteins of the same taxonomic group as the study data to account for selection pressures that are appropriate for the study data. Next, the selection of the best-fitting substitution models for a given dataset can be performed using a variety of frameworks (e.g. [20, 23, 52–54]). However, the results provided by these frameworks should be carefully evaluated in terms of biological considerations. For instance, using a framework for substitution model selection, some authors [51] found that the best-fitting empirical substitution model for datasets from Proteobacteria and Archaea was a model inferred from retroviral *Pol* proteins, which is unlikely to accurately describe the evolutionary processes of these datasets. The limited number of available empirical substitution models of protein evolution, especially those implemented in phylogenetic frameworks, can complicate the identification of appropriate substitution models for specific datasets. Indeed, there is a need for the development of additional substitution models of protein evolution.

In another experiment, we assessed the folding stability of proteins derived from modeling with different empirical substitution models. We found that all empirical substitution models produce protein sequences that are less stable than the real protein sequences. However, for some protein families, this effect was partially reduced when applying the best-fitting empirical substitution model. These findings indicate two important concerns for modeling protein evolution. First, empirical substitution models are inadequate for producing proteins with realistic folding stability. This is expected, as these models do not incorporate direct constraints from the protein structure and usually assume a single exchangeability matrix for all protein sites, leading to unrealistic modeling in this context [55]. To address this, one may consider applying structurally constrained substitution models that outperformed empirical substitution models [19, 25, 43], although, unfortunately, these models have been implemented in only a few phylogenetic frameworks due to their mathematical complexity [19, 56]. Second, if empirical substitution models must be used, applying the best-fitting empirical substitution model based on phylogenetic likelihood (including heterogeneity of the substitution rate among sites, if present) is recommended, as it could partially reduce the bias of unrealistic folding instability. Indeed, previous studies have recommended applying the best-fitting substitution model for accurate phylogenetic tree and ancestral sequence reconstructions, particularly when there is sufficient molecular diversity in the study dataset to allow the model to make crucial evolutionary decisions that can be reflected in the inferences [4, 5]. Therefore, while empirical substitution models are not the best choice for studying certain aspects such as the folding stability of the modeled proteins, in the absence of more accurate alternatives (e.g. structurally constrained substitution models), using a best-fitting substitution model based on likelihood can be preferable to relying on an arbitrary empirical substitution model.

Altogether, we provide a freely accessible and user-friendly database of empirical substitution models of protein evolution, designed to facilitate access to this scientific knowledge, which is fundamental for likelihood-based protein phylogenetic methods. Additionally, we assess the empirical substitution models in terms of their similarity in evolutionary patterns and the folding stability of the modeled proteins. We also provide recommendations for selecting among empirical substitution models, which can be especially useful when a best-fitting model is unavailable for evolutionary inferences.

## Supplementary Material

baaf052_Supplemental_File

## Data Availability

The database and the associated framework *EModelDB* are freely available at https://github.com/Paula-Iglesias-Rivas/EModelDB. The real and simulated data used for assessing the empirical substitution models can be accessed from the Zenodo repository at https://doi.org/10.5281/zenodo.14960165.
