# Supplementary material for: Empirical substitution models of protein evolution: database, relationships, and modeling considerations
Source: Database (Oxford). 2025 Sep 24;2025:baaf052. doi: 10.1093/database/baaf052 (PMC12462380; doi:10.1093/database/baaf052)

Supplementary Material

Empirical substitution models of protein evolution: Database, relationships and modeling considerations

The supplementary material includes the Figures S1-S2.

Figure S1. Agglomerative clustering of common empirical substitution models of protein evolution according to the matrix of relative substitution rates among amino acids. The normalized matrix of relative substitution rates among amino acids was applied to determine the distance between every pair of commonly used substitution models. A bottom-up agglomerative clustering method, neighbor joining, was then applied. Each model was color-coded based on the type of proteins used to construct the model: mitochondrial proteins (blue), chloroplast proteins (green), virus proteins (red), and nuclear proteins (general in purple and taxon-specific in pink). The cluster based on the matrix of relative substitution rates among amino acids and amino acid frequencies at equilibrium is presented in Figure 2, and the cluster based on the amino acid frequencies at equilibrium is presented in Figure S2.

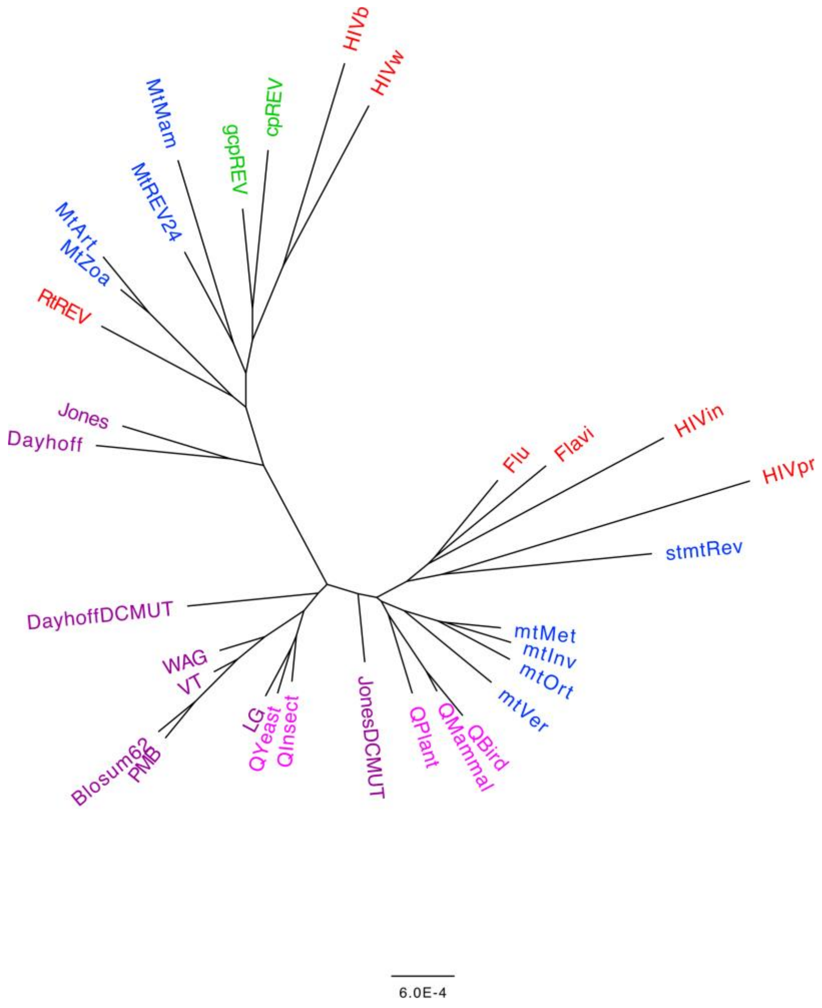

Figure S2. Agglomerative clustering of common empirical substitution models of protein evolution according to the amino acid frequencies at equilibrium. The amino acid frequencies at equilibrium were used to determine the distance between every pair of commonly used substitution models. A bottom-up agglomerative clustering method, neighbor joining, was then applied. Each model was color-coded based on the type of proteins used to construct the model: mitochondrial proteins (blue), chloroplast proteins (green), virus proteins (red), and nuclear proteins (general in purple and taxon-specific in pink). The cluster based on the matrix of relative substitution rates among amino acids and amino acid frequencies at equilibrium is presented in Figure 2, and the cluster based on the relative substitution rates among amino acids is presented in Figure S1.

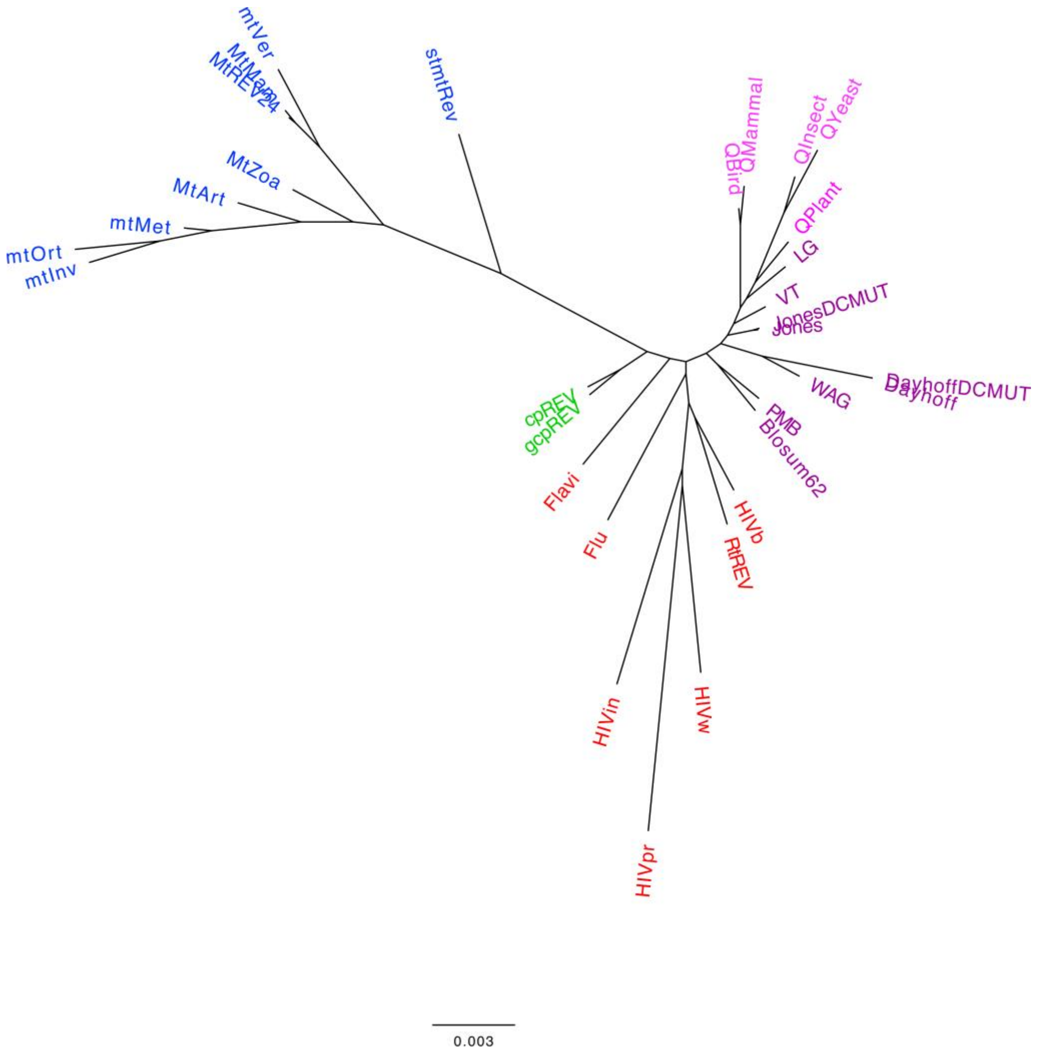

Supplement: baaf052_Supplemental_File [file baaf052_supplemental_file.pdf]
